# Does low confidence drive reliance on AI, or does reliance erode confidence? A four-wave RI-CLPM study of chinese language and literature undergraduates

**DOI:** 10.1371/journal.pone.0361489

**Published:** 2026-10-26

**Authors:** Xinran Du, Sedan Yu

**Affiliations:** 1 Department of Chinese Language and Literature, Hong Kong Metropolitan University, Hong Kong SAR, China; 2 University of Leeds, Leeds, United Kingdom; Bahir Dar University, ETHIOPIA

## Abstract

Generative AI tools are now routinely used by Chinese Language and Literature majors for tasks once considered the province of individual literary craft: drafting verse, shaping narrative prose, revising fiction. Two competing accounts describe what this means for students’ confidence in their own creative writing. A cognitive-offloading account holds that repeated reliance on AI displaces the mastery experiences through which writing self-efficacy is built, so reliance erodes confidence over time. A compensatory-use account holds the reverse: students whose confidence is already low turn to AI to avoid the discomfort of unassisted composition, so low confidence drives reliance. Cross-sectional and standard cross-lagged designs cannot separate these accounts because they confound stable between-person differences with genuine within-person change. We addressed this using a random-intercept cross-lagged panel model (RI-CLPM) across four waves spanning one academic year among Chinese Language and Literature undergraduates (*N* = 390 recruited; *N* = 387 analyzed under full-information maximum likelihood, of whom 298 supplied complete four-wave data). Generative AI writing reliance (GAWR) and literary creative writing self-efficacy (LCWSE) were assessed at each wave. The trait-level association between the two constructs was negative and could not be distinguished from zero (*r* = −.342, Δχ²(1) = 2.26, *p* = .133). At the within-person level, declines in self-efficacy predicted subsequent increases in reliance (β = −.276, *p* < .001). The reverse path, from reliance to subsequent self-efficacy, was smaller and fell short of the conventional threshold (β = −.085, *p* = .072). We state explicitly that this second result is not interpretable as evidence against erosion: a Monte Carlo power analysis put this design's power to detect an effect of that magnitude at 53.2%, close to a coin flip, so the erosion pathway is undetermined in these data and this null supplies no basis for concluding that erosion does not operate. A direct comparison of the two cross-lagged paths favored compensation (Δχ²(1) = 8.83, *p* = .003; bootstrap 95% CI for the difference [.020, .218] across 5,000 resamples), with the qualification that this comparison sets an adequately powered estimate against an underpowered one and therefore describes what this design was able to detect. Compensation is the pathway this study establishes; whether reliance also erodes confidence over a longer horizon is an open question. We discuss implications for writing pedagogy in Chinese Language and Literature programs and for how underpowered null results should be interpreted in AI-in-education panel research.

## 1. Introduction

Generative AI writing tools have moved past grammar correction and citation formatting into the imaginative core of literary composition: plot outlines, opening lines, full paragraphs of descriptive prose that a student can lightly edit and submit as her own. Chinese Language and Literature programs, which train the next generation of writers, editors, and literature teachers, have had to absorb this shift with almost no longitudinal evidence about what it is doing to students’ confidence in their own creative capacities. The question this paper asks is not whether students use these tools, they clearly do, but which way the causal arrow runs between using them and feeling capable without them.

Two theoretical accounts offer opposite predictions. The first draws on cognitive offloading theory [1], which holds that when an external tool can perform a cognitive task, people redirect effort away from internal processing and toward the tool. Applied to writing, repeated delegation of idea generation and phrasing to AI would substitute for the mastery experiences through which writing self-efficacy is built [2,3]. Under this account, reliance on generative AI should erode students’ confidence in unassisted literary composition over time, an “erosion” pathway consistent with a wave of recent work on AI-linked cognitive offloading and metacognitive laziness [4–6]. The second account inverts the causal arrow. Drawing on the compensatory internet use tradition [7], it holds that people turn to external tools not because tools erode capacity but because a prior deficit, low confidence, high anxiety, a felt inability to produce competent original work, makes the unassisted task aversive. Under this “compensation” account, a decline in creative writing self-efficacy should precede and predict increased reliance on AI, not the other way around.

This distinction is not academic hairsplitting. If reliance erodes confidence, the appropriate institutional response is to constrain or scaffold AI use in creative writing courses so that students still accumulate mastery experiences. If declining confidence drives reliance, constraining AI access without addressing the underlying confidence deficit is unlikely to help, and may simply remove a coping tool while leaving the cause untouched. Choosing between these responses requires knowing which direction of influence actually operates, and existing research cannot yet answer that question, because it has not used a design capable of separating two things that are easy to confuse: whether a student who is generally more AI-reliant is also generally less confident (a stable difference between students), and whether a given student's own reliance and confidence rise and fall together over time in a predictable order (a moving relationship within a student). The method we use in this paper, the random-intercept cross-lagged panel model, exists specifically to keep those two questions from being answered as if they were one.

The empirical literature on generative AI and writing self-efficacy has grown quickly [8–10], including several studies conducted with Chinese university students specifically [11–13]. Almost all of it, however, is cross-sectional or relies on a single measurement occasion, which means it can describe association but not sequence. The handful of longitudinal exceptions in adjacent domains, such as Zhou et al.’s [14] three-wave study of compensatory social networking use, employ standard cross-lagged panel models that conflate two conceptually distinct sources of covariation: the possibility that people who are generally more reliant on AI also tend to be generally less confident (a stable, trait-like association) and the possibility that a person's own fluctuations in reliance genuinely precede or follow that same person's fluctuations in confidence (a within-person, state-like process). Hamaker et al. [15] showed that standard cross-lagged models systematically confound these two sources, and the random-intercept cross-lagged panel model, or RI-CLPM, was developed specifically to separate them [16]. No study we identified has applied this design to the relationship between generative AI writing reliance and writing self-efficacy, and none has done so in the literary, rather than academic or expository, writing context that defines Chinese Language and Literature education, a context in which originality and personal voice are the explicit object of instruction and therefore, plausibly, the domain in which AI delegation carries the most consequence.

The present study addresses this gap. We surveyed Chinese Language and Literature undergraduates at four occasions across one academic year, measuring generative AI writing reliance (GAWR) and literary creative writing self-efficacy (LCWSE) at each wave, and modeled their relationship using RI-CLPM. This design lets us ask, and separately answer, three questions that prior designs collapse into one: whether students who are generally more AI-reliant are also generally less confident (a between-person question), whether within-person increases in reliance precede within-person decreases in confidence or the reverse (a within-person, directional question), and, if one pathway is detectable and the other is not, whether that asymmetry reflects a real difference in mechanism strength or simply a difference in how much statistical power this design had to find each one. We derive competing predictions from cognitive offloading theory and from the compensatory internet use tradition and test both directions in the same model, which, to our knowledge, has not been done for this pair of constructs or for this population. We do not claim to have merged these two literatures into a new theory; we use them to generate two testable, opposing directional predictions and let the four-wave design adjudicate between them as far as the data allow.

The remainder of the paper proceeds as follows. Section 2 develops the dual-pathway theoretical framework and derives four hypotheses. Section 3 describes the four-wave design, measures, and analytic strategy. Section 4 reports the RI-CLPM results. Section 5 discusses the findings’ theoretical and pedagogical implications, along with the study's limitations.

## 2. Literature review and hypotheses

### 2.1 Generative AI in literary and creative writing education

Research on generative AI and student writing has expanded rapidly, but its center of gravity sits firmly in academic and expository genres: essay structure, argumentative coherence, English-as-a-foreign-language proficiency [8,10]. Li and Li [17] and G. Zhang and Yu [18] both link generative AI use to self-efficacy more broadly, the former across college students in general and the latter specifically through generative AI trust and risk perception, but neither examines writing, let alone literary writing, as the domain of self-efficacy at stake. Pellas [9] found that undergraduates using generative AI platforms for narrative tasks reported gains in narrative intelligence alongside shifts in writing self-efficacy, but the design was cross-sectional and the narrative task was instructional rather than literary in the sense of original creative production. Yeung's [19] study of AI-supported automated writing evaluation feedback and H. Zhang et al.’s [20] study linking generative AI feedback to writing engagement both examined revision and feedback processes rather than the generative, idea-originating use that most concerns literature educators. A parallel body of work locates writing self-efficacy in the conditions of instruction that surround the tool. Talebzadeh et al. [21] compared flipped, online, and in-person delivery of writing instruction and found that modality itself shapes learners’ writing motivation and writing self-efficacy trajectories, and Ahangaran and Pouyan [22] show that integrating automated corrective feedback into ESP writing instruction changes how learners engage with production tasks. Both results indicate that confidence in writing is responsive to the instructional environment in which a tool is encountered, which bears directly on the compensation account developed below: if a confidence deficit is what draws students toward AI, the conditions under which that deficit forms are part of the mechanism and not merely its backdrop. Among Chinese samples specifically, Liu [12] reported associations between AI writing tool use and both writing quality and self-efficacy among Chinese university students, Chen et al. [11] surveyed AI literacy for academic English writing, and Shin [13] argued, on largely conceptual grounds, that AI literacy should be integrated into Chinese Language and Literature curricula. None of this work isolates literary or creative composition, the genre in which personal voice, originality, and aesthetic judgment, rather than argumentative correctness, are the explicit criteria of success, and none of it is longitudinal in a way that permits causal ordering. Lin and Wang [23] came closest, showing that graduate students’ self-efficacy, time pressure, and trust jointly predicted generative AI use in thesis writing, but the design was again cross-sectional and the writing task was academic rather than literary.

This matters because literary creative writing is arguably the genre in which AI delegation carries the highest theoretical stakes. Academic writing has agreed-upon structures that a novice can learn by imitation without much loss of “voice,” because voice is not the point. Literary writing has no such external criterion; the writer's distinctive way of seeing is constitutive of the product, not incidental to it. If reliance on AI substitutes for the practice through which a personal creative voice develops, or if declining confidence in one's own voice pushes a writer toward AI-generated substitutes, the consequences plausibly run deeper than in genres where competent imitation suffices. Chinese Language and Literature programs, which exist specifically to cultivate this kind of literary competence, are therefore a theoretically apt and empirically neglected setting in which to study the relationship.

### 2.2 Cognitive offloading and the erosion pathway

Cognitive offloading theory [1] describes a general tendency to shift cognitive effort onto external resources whenever those resources are available and reliable, from writing a note to avoid remembering a phone number to rotating a physical object rather than mentally rotating its image. Dunn and Risko's [24] metacognitive account adds that this shift is not merely a passive default but an outcome of an ongoing, if often implicit, cost-benefit judgment about whether internal or external processing is more efficient in the moment. Generative AI is, in this framework, simply a very capable and very available offloading target, and a fast-growing body of work has begun documenting offloading onto AI specifically: García-Barrios [25] frames the phenomenon as a tension between AI as an “epistemic partner” and AI as a “cognitive crutch,” Jain et al. [5] report associations between AI-assisted task performance and reduced critical thinking engagement, and Zahid et al. [6] link generative AI chatbot use in higher education to patterns resembling addiction alongside cognitive offloading. Dizon et al. [4] operationalize a closely related construct, metacognitive laziness, as a direct consequence of AI-driven offloading, and Yurt and Kuşci [26] show that epistemic laziness and metacognitive weakness mediate the relationship between AI use and critical thinking. We note that this entire body of evidence concerns AI offloading's effects on critical thinking and metacognition generally; none of it measures writing self-efficacy directly, so the extension to literary creative writing self-efficacy below is an inference by analogy from an adjacent domain, not a direct empirical finding.

The mechanism by which offloading would erode writing self-efficacy specifically follows from Bandura's [2,27] social cognitive account of self-efficacy, in which the strongest source of efficacy belief is mastery experience: the repeated, effortful accomplishment of the task itself. Pajares's [3] review of writing self-efficacy applies this directly, arguing that confidence in one's writing is built through cumulative, self-generated success at composing text, not through observing or lightly editing someone else's output. If a student routinely delegates the generative core of a creative writing task, the plot decision, the opening line, the descriptive turn of phrase, to an AI system, the occasions on which mastery experience would normally accrue are systematically reduced. Confidence, on this account, does not merely fail to grow; it should erode relative to where it would otherwise be, because the felt experience of “I did this myself” becomes rarer even as output continues to be produced.

This leads to our first substantive, directional hypothesis, framed at the level of within-person change rather than between-person description:

**H2 (Erosion pathway).** Within-person increases in generative AI writing reliance (GAWR) at a given wave will negatively predict within-person literary creative writing self-efficacy (LCWSE) at the subsequent wave, controlling for each construct's own prior level and for stable between-person differences.

### 2.3 Compensatory internet use and the compensation pathway

A second, less intuitive but equally well-grounded literature suggests the causal arrow might run the other way. Kardefelt-Winther's [7] compensatory internet use framework was developed as a corrective to research that treated heavy technology use as an intrinsically pathological driver of poor psychological outcomes; Kardefelt-Winther argued instead that much technology use is better understood as a coping response to a pre-existing psychological need or deficit, motivation that flows from the deficit to the behavior, not the reverse. Wegmann and Brand's [28] work on internet-communication disorder extends this logic to social and coping motives specifically, and Zhou et al.’s [14] three-wave panel study is a direct empirical demonstration that compensatory technology use can be modeled longitudinally: they found that compensatory social networking site use and depressive symptoms were entangled in ways a single cross-sectional snapshot could not disentangle. Lin [29] reports a parallel pattern for avoidance and emotion-focused coping as a mediator of problematic internet use, reinforcing that turning to a technological substitute is frequently a response to an aversive internal state rather than its cause.

Applied to creative writing, the compensatory account holds that a student who doubts her own capacity to produce a good opening line, or who has recently received discouraging feedback on a piece of fiction, experiences the blank page as more aversive than a student with high self-efficacy does. Generative AI offers an immediate way to avoid that aversive state: it produces usable text without requiring the student to confront her own perceived inadequacy. On this account, declining confidence precedes and causes increased reliance, not the reverse. Several recent studies on AI-specific dependence measures are consistent with a motivational, deficit-driven account of reliance, even though most were not designed to test directionality: Wei et al.’s [30] AIED scale and Akbari et al.’s [31] AIUMS/PGAU framework both treat problematic generative AI use as arising from underlying emotional or motivational needs, and Yao et al. [32] explicitly link self-esteem, a construct closely related to domain-specific self-efficacy, to problematic AI chatbot use through psychological mediators.

This yields our second directional hypothesis. Because lower confidence is expected to predict higher subsequent reliance, the corresponding path coefficient is expected to be negative in sign, exactly as in H2, even though the underlying relationship (less confidence, more reliance) runs in the substantively opposite direction from erosion (more reliance, less confidence):

**H3 (Compensation pathway).** Within-person decreases in literary creative writing self-efficacy (LCWSE) at a given wave will predict within-person increases in generative AI writing reliance (GAWR) at the subsequent wave, controlling for each construct's own prior level and for stable between-person differences.

### 2.4 Trait-state separation and the case for RI-CLPM

H2 and H3 are stated as within-person, state-level claims because the erosion and compensation mechanisms they describe are inherently about change within a person over time, not about who a person generally is. Yet nearly all existing evidence on AI reliance and writing confidence is between-person: it compares students who differ in reliance to students who differ in confidence, without following the same students as either construct changes. This distinction is not a minor statistical nicety. Hamaker et al. [15] demonstrated that the standard cross-lagged panel model, which has been the default longitudinal tool in this literature, implicitly assumes that all individuals share the same trait-like mean level of each construct, an assumption that is almost never true and that, when violated, causes the model to misattribute stable between-person differences to within-person causal processes. A negative cross-lagged path in a standard CLPM could reflect a genuine within-person process, or it could simply reflect the fact that students who are chronically more reliant on AI also happen to be chronically less confident, with neither fluctuation actually driving the other over time.

The random-intercept cross-lagged panel model addresses this by explicitly partitioning each construct's variance into a stable, person-specific random intercept and a wave-specific within-person deviation from that person's own average, and then estimating autoregressive and cross-lagged paths only among the within-person deviations [16]. This partition allows a between-person, trait-level question, do chronically higher-reliance students also tend to be chronically lower-confidence students, to be asked and answered separately from the within-person, state-level questions posed in H2 and H3. We therefore include a between-person hypothesis about the random intercepts themselves:

**H1 (Trait-level association).** The random intercepts of GAWR and LCWSE, representing each student's stable, wave-invariant tendency toward AI reliance and toward creative writing self-efficacy respectively, will be negatively correlated: students who are generally more AI-reliant across the academic year will tend to be generally less confident in their own literary creative writing.

We note that Lüdtke and Robitzsch [33] and Zheng and Valente [34] have each raised methodological concerns about the precision with which RI-CLPM estimates random-intercept covariances and cross-lagged paths, particularly with a limited number of waves; we return to this critique when interpreting our results, since it bears directly on how confidently H1 in particular can be evaluated with four waves of data.

### 2.5 Comparing the two pathways

H2 and H3 are not mutually exclusive. Cognitive offloading and compensatory use describe genuinely different mechanisms, and both could operate simultaneously within the same students, one process gradually eroding confidence through reduced mastery experience while the other pulls low-confidence moments toward greater reliance. Zhao et al. [35] applied both a standard CLPM and an RI-CLPM to a different substantive question (harsh parenting, shyness, and cyber victimization) and found that conclusions about the relative strength of two competing directional paths shifted once trait-level confounding was removed, which is the precedent for treating a direct comparison between H2 and H3 as answerable, rather than merely descriptive, once trait and state are separated.

We offer a tentative reason to expect the two pathways to differ in strength within a one-year window, while flagging it explicitly as an auxiliary conjecture rather than a load-bearing derivation from either theory: cognitive offloading, as we have described it above, is a cumulative-displacement mechanism, and nothing in the theory specifies how much cumulative exposure is required before it produces a measurable confidence deficit. A compensatory response to felt inadequacy plausibly requires no comparable accumulation; it can, in principle, follow a single discouraging episode. If that asymmetry in required dosage is real, the compensation pathway would be easier to detect than the erosion pathway within a single academic year, independent of whether the two mechanisms are equally real or equally strong in the long run. We treat this as a hypothesis about which pathway this specific design can detect, not a settled claim about which mechanism is fundamentally stronger, and we return to the difference between those two things when interpreting the results.

**H4 (Relative pathway magnitude).** The magnitude of the compensation pathway (H3, LCWSE → GAWR) will exceed the magnitude of the erosion pathway (H2, GAWR → LCWSE) as estimated within this one-year, four-wave window.

H1 through H4 test two independently derived directional predictions, cognitive offloading's erosion pathway and compensatory internet use's compensation pathway, against each other in a single design that separates trait-level covariation (H1) from within-person, directional processes (H2, H3), and asks which within-person process this design can detect (H4). We do not treat H4's confirmation or disconfirmation as adjudicating which theory is correct in general; a design with different power characteristics, most plausibly a longer panel, could detect a different pattern without either theory being wrong. We flag one further consequence of treating H4 as a hypothesis rather than as a purely exploratory comparison: if the dosage asymmetry we conjecture above is real, it implies in advance that this design would have unequal power to detect the two paths, which means H4 is not necessarily a fair head-to-head test even in principle, a point we return to when interpreting the results. Fig 1 depicts the full model (Table 1).

**Table 1:** Standardized parameter estimates for the time-invariant random-intercept cross-lagged panel model (FIML, N  =  387)).

Parameter	Estimate	SE	p
Autoregressive paths			
GAWR(t-1) → GAWR(t)	.324	.056	< .001
LCWSE(t-1) → LCWSE(t)	.421	.054	< .001
Cross-lagged paths			
GAWR(t-1) → LCWSE(t) [H2, erosion]	−.085	.048	.072
LCWSE(t-1) → GAWR(t) [H3, compensation]	−.276	.050	< .001
Random intercept (co)variances			
Var(RI-GAWR)	.216	.048	< .001
Var(RI-LCWSE)	.211	.063	< .001
Cov(RI-GAWR, RI-LCWSE) [H1]	−.073	.043	.093
Corr(RI-GAWR, RI-LCWSE) [H1]	−.342	.154	.133
Within-person residual variances			
Var(wGAWR2)	.360	.035	< .001
Var(wGAWR3)	.337	.033	< .001
Var(wGAWR4)	.350	.034	< .001
Var(wLCWSE2)	.508	.048	< .001
Var(wLCWSE3)	.426	.039	< .001
Var(wLCWSE4)	.428	.041	< .001
Model fit			
χ² (df)	7.44 (17)		.977
CFI / TLI	1.000 / 1.016		
RMSEA / SRMR	.000 / .021		

*Note. GAWR = Generative AI Writing Reliance; LCWSE = Literary Creative Writing Self-Efficacy; RI = random intercept; w = within-person component. Autoregressive and cross-lagged paths were constrained equal across the three wave-to-wave transitions; within-person residual variances were freely estimated at each wave (not constrained equal). Composite-score residual terms were fixed to zero and the random intercepts were constrained orthogonal to the Wave-1 within-person components. N = 387 (all baseline respondents). Estimation: R 4.6.1, lavaan 0.7−2, full-information maximum likelihood. The H1 row is reported in both covariance and correlation metric. Path coefficients and the correlation are standardized; variances and the covariance are unstandardized. All p-values are Wald tests of the unstandardized parameters, except in the correlation row, which reports the likelihood-ratio test of the covariance, Δχ²(1) = 2.26, p = .133. Complete-case estimates (n = 298) for the four structural paths were .323, .412, −.100, and −.275 respectively.*

**Fig 1:**
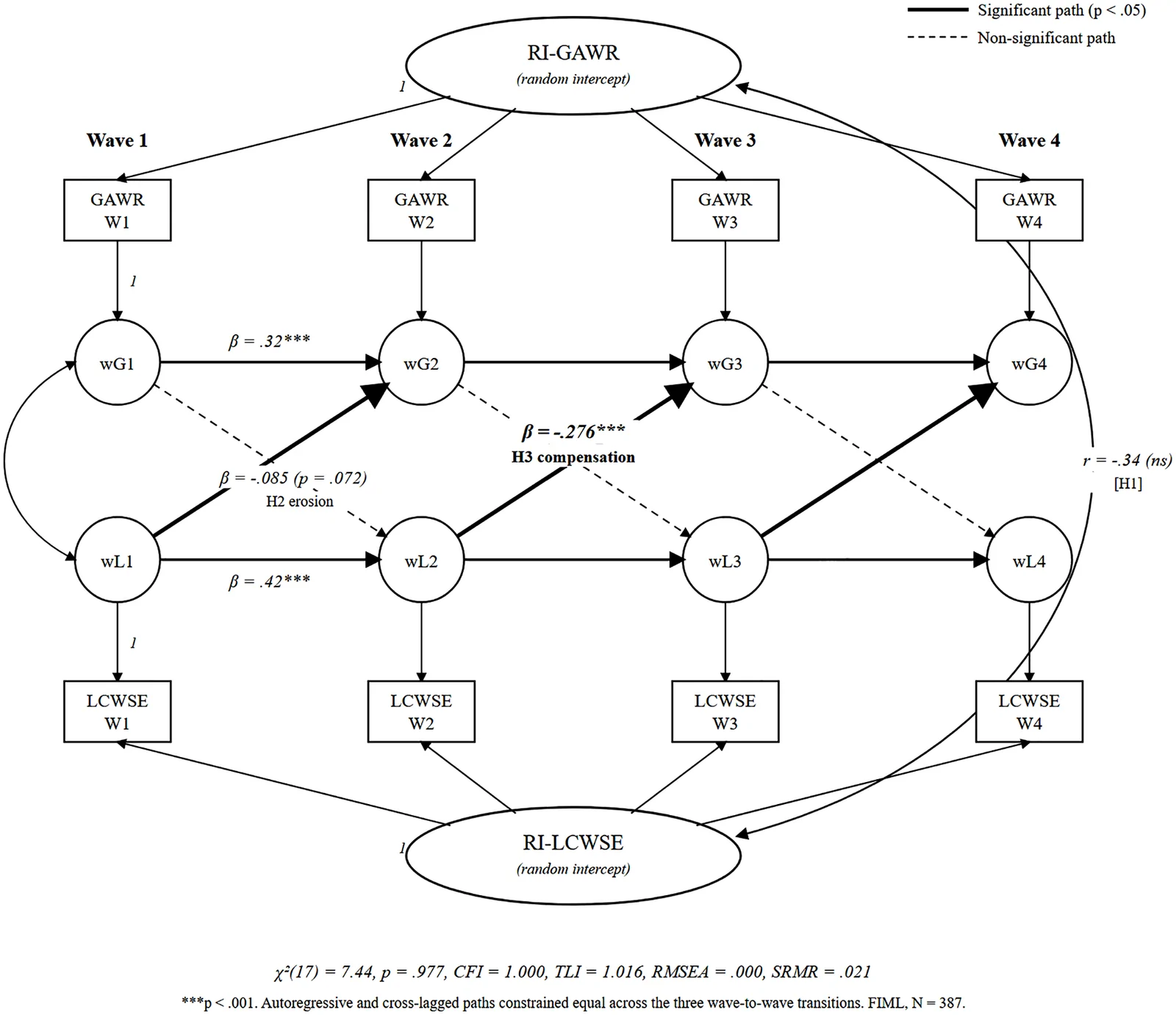
Random-intercept cross-lagged panel model for generative AI writing reliance (GAWR) and literary creative writing self-efficacy (LCWSE) across four waves. Rectangles are observed composite scores, circles are wave-specific within-person components, and ellipses are the two random intercepts. Coefficients are standardized estimates from the time-invariant model fitted by full-information maximum likelihood to all 387 baseline respondents (Table 1); autoregressive and cross-lagged paths were constrained equal across the three wave-to-wave transitions. Bold solid lines mark the compensation pathway (H3), dashed lines the erosion pathway (H2). ***p < .001.

## 3. Methodology

### 3.1 Design and procedure

We conducted a four-wave longitudinal panel survey spanning one academic year, with waves anchored to fixed points in the academic calendar: Wave 1 in the second week of the spring semester (February 2025), Wave 2 at the spring semester's midpoint (April 2025), Wave 3 at the start of the fall semester (September 2025), and Wave 4 at the fall semester's midpoint (November 2025). The resulting intervals are unequal. Wave 1 to Wave 2 and Wave 3 to Wave 4 each span approximately nine weeks of term time, while Wave 2 to Wave 3 spans the summer vacation and runs to approximately 21 weeks. Anchoring the waves to comparable points within each semester holds the instructional context constant across occasions, and every interval is long enough for meaningful within-person change in both constructs to occur. Because the equality constraint imposed on the structural paths (Section 3.4) assumes the lagged process operates comparably across all three transitions, we test that assumption directly by freeing the Wave 2 to Wave 3 transition and comparing model fit (Section 4.2). Data were collected through an anonymous online survey administered via each participating institution's course management platform; a unique, self-generated anonymous identifier allowed responses to be linked across waves without collecting identifying information. This study was approved by the Ethics Committee of the Hangzhou Educational Science Research Institute prior to data collection (Approval No. HZESRI-923; approved 20 November 2024) and was conducted in accordance with the Declaration of Helsinki. Participants were recruited between 24/02/2025 and 14/03/2025; longitudinal data collection across all four waves spanned from 24/02/2025 to 21/11/2025. All participants provided informed consent electronically at Wave 1 prior to completing any survey items. Participants were informed that participation was entirely voluntary, that they could withdraw at any time without penalty, and that their responses would be kept anonymous. No identifying information was collected; a unique self-generated anonymous identifier linked each participant’s responses across waves.

### 3.2 Participants

Participants were undergraduate students (second through fourth year) enrolled in Chinese Language and Literature programs at three universities in eastern China varying in selectivity, recruited via course instructors in departments offering creative writing or literary composition coursework. We recruited 390 students at Wave 1. During data screening, three respondents were removed for evident careless responding at baseline, identified by near-zero variance across the full set of Wave 1 items (standard deviation < 0.15), leaving a baseline analytic sample of 387. Retention was 92.0% at Wave 2, 85.3% at Wave 3, and 77.0% at Wave 4 relative to the baseline sample, yielding 298 participants with complete data across all four waves. Missingness is monotone: 31 participants provided Wave 1 only, 26 provided Waves 1 and 2, 32 provided Waves 1–3, and 298 provided all four waves. Attrition was examined against Wave-1 GAWR and LCWSE scores, against each of the five Wave-1 covariates, and against each participant's Wave 1 to Wave 2 change on both constructs; all three analyses are reported in Section 4.1. The structural RI-CLPM is estimated by full-information maximum likelihood on all 387 baseline respondents, and the complete-case subsample (*n* = 298) is reported alongside it as a robustness check. Sample characteristics at Wave 1 are given in Table 2.

**Table 2:** Wave-1 sample characteristics by four-wave completion statusstatus.

Characteristic	Full sample (N = 387)	Four-wave completers (n = 298)	Non-completers (n = 89)
Gender, n (%)			
Female	209 (54.0)	160 (53.7)	49 (55.1)
Male	178 (46.0)	138 (46.3)	40 (44.9)
Academic year, n (%)			
Second year	151 (39.0)	109 (36.6)	42 (47.2)
Third year	137 (35.4)	113 (37.9)	24 (27.0)
Fourth year	99 (25.6)	76 (25.5)	23 (25.8)
Literature GPA, M (SD)	3.37 (0.36)	3.36 (0.37)	3.41 (0.35)
General AI familiarity, n (%)			
1 (least familiar)	70 (18.1)	59 (19.8)	11 (12.4)
2	76 (19.6)	56 (18.8)	20 (22.5)
3	85 (22.0)	60 (20.1)	25 (28.1)
4	78 (20.2)	62 (20.8)	16 (18.0)
5 (most familiar)	78 (20.2)	61 (20.5)	17 (19.1)
Creative-writing course load, M (SD)	1.94 (1.35)	1.93 (1.38)	1.98 (1.25)
GAWR Wave 1, M (SD)	2.96 (0.84)	2.97 (0.84)	2.94 (0.84)
LCWSE Wave 1, M (SD)	3.22 (0.90)	3.23 (0.92)	3.20 (0.82)

*Note. GAWR = Generative AI Writing Reliance; LCWSE = Literary Creative Writing Self-Efficacy. All characteristics were collected at Wave 1. Percentages are column percentages within each category block. Completers and non-completers did not differ on any characteristic (all p > .27; see Section 4.1), and a logistic regression predicting dropout from all seven Wave-1 variables jointly did not improve on an intercept-only model, χ²(7) = 2.76, p = .906.*

**Sample size.** The Wave-1 recruitment target of 390 was set to retain approximately 300 participants after the attrition anticipated across four waves, a figure chosen with reference to the sample sizes at which RI-CLPM parameter recovery has been reported as acceptable for panels of four or more waves. We did not conduct a formal a priori power analysis for each of the four hypotheses before data collection, and the head-to-head framing of H4 should be read in that light: the design was sized for the panel model as a whole and was not calibrated in advance to detect the two cross-lagged paths with equal sensitivity. Section 4.3 reports a sensitivity analysis across a range of sample sizes that gives the effect magnitudes this design could and could not detect.

### 3.3 Measures

**Generative AI Writing Reliance (GAWR).** GAWR was measured with six items adapted from recent generative-AI dependence and problematic-use scales [30,31,36], selected for their emphasis on task dependence and reduced autonomy rather than general technology attitudes, and reworded to reference literary and creative writing tasks specifically (e.g., “I rely on AI tools to generate ideas for my literary writing assignments”). The construct is defined at the level of task delegation: the degree to which a student's literary writing output depends on generative AI for work the student would otherwise perform. It is kept separate from general AI use, a frequency-of-contact variable that carries no implication of delegation and that we measured independently as AI familiarity, and from AI dependence in the clinical sense, which the source scales operationalize through distress, loss of control, and functional impairment. Items indexing affective attachment or withdrawal were not carried over; each of the six retained items asks about substituting AI output for the student's own generative work in a literary writing task, which is the quantity on which both the offloading and the compensation accounts make predictions. Items used a 5-point Likert response format (1 = strongly disagree to 5 = strongly agree) and were administered identically at all four waves. Internal consistency was excellent at every wave (Cronbach's α = .930 to .935; McDonald's ω = .933 to .937).

**Literary Creative Writing Self-Efficacy (LCWSE).** LCWSE was measured with seven items adapted from the Pajares writing self-efficacy tradition [37–39] together with items drawn from Graham and MacArthur's [40] Student Writing Ability Self-Efficacy Scale, Locke and Johnston's [41] self-efficacy scale for the teaching of writing, and Doenyas et al.’s [42] non-Western adaptation of a situated academic writing self-efficacy measure, recontextualized from academic to literary genres (poetry, prose, and short fiction). Because no existing instrument measures self-efficacy for literary composition, items were drawn from several sources and harmonized against a single criterion: each candidate item had to ask about confidence in executing a self-generated writing task, which is the sense of efficacy Bandura [2] and Pajares [3] identify as built from mastery experience. Items concerning writing-related outcomes such as grades or teacher approval, items concerning self-regulation of the writing process, and items concerning the teaching of writing to others were excluded under that criterion; Locke and Johnston's [41] scale contributed only items phrased as personal writing capability, and Doenyas et al.’s [42] adaptation contributed the situated phrasing that ties each item to a concrete task. Every retained item was then rewritten to name a literary genre in place of the academic genre of the source. A sample item is “I am confident I can write an original short story that expresses my own voice.” Items used the same 5-point Likert format and were administered at all four waves. Internal consistency was excellent at every wave (Cronbach's α = .946 to .952; McDonald's ω = .948 to .954).

**Covariates.** Gender, academic year, self-reported GPA in literature coursework, general AI familiarity (to distinguish AI-specific creative reliance from generic technology fluency), and self-reported creative-writing course load were collected at Wave 1 for sample description and are reported in Table 2; because RI-CLPM already partitions stable between-person differences into the random intercept, these covariates were not entered as time-varying predictors in the primary structural model. They are used in the attrition analysis reported in Section 4.1.

### 3.4 Analytic strategy

**Preliminary analyses.** We computed descriptive statistics and zero-order correlations among all wave-specific composite scores. We evaluated the measurement model with confirmatory factor analysis at Wave 1 to establish that GAWR and LCWSE items loaded on separate, well-fitting factors, and report Wave-1 standardized item loadings alongside this model. We additionally tested configural and metric measurement invariance of both factors across all four waves using two parcels per construct per wave (items 1−3 and items 4–6/4–7 averaged into two indicators each), which keeps the invariance model numerically tractable while still testing whether the items mean the same thing at every wave. Internal consistency was indexed by Cronbach's α and by McDonald's ω, the latter because ω does not assume equal item-factor loadings and is the more appropriate index for a newly assembled composite. Attrition was examined in three ways: univariate comparisons of four-wave completers and non-completers on Wave-1 GAWR, Wave-1 LCWSE, and each of the five Wave-1 covariates; a logistic regression predicting dropout from all seven variables jointly; and, among participants who supplied at least Waves 1 and 2, a logistic regression predicting later dropout from each participant's Wave 1 to Wave 2 change on both constructs. Common method bias, an especially relevant concern given that both constructs were self-reported at each occasion, was addressed both procedurally, following Podsakoff et al.’s [43,44] recommendations (anonymous administration, assurance of no correct answers, and physical separation of the two item sets within the survey), and with a single-factor comparison model (all Wave-1 GAWR and LCWSE items loading on one factor) as a baseline check; we did not rely on this single-factor test alone, since Podsakoff et al. [44] note it can itself be uninformative when applied as the sole safeguard.

**RI-CLPM specification.** The structural model followed the standard random-intercept cross-lagged panel model specification [15,16]. Each wave's observed GAWR and LCWSE composite score was decomposed into a random intercept, common to all four waves and capturing the person's stable trait-like level, and a wave-specific within-person component representing that wave's deviation from the person's own trait level; loadings from each random intercept and each within-person component to their respective observed wave scores were fixed to 1, so no additional measurement-error term was estimated for the composite scores themselves. The two random intercepts were allowed to correlate (testing H1) and were constrained orthogonal to the Wave-1 within-person components, which the standard specification requires for the trait-state decomposition to be identified. Within-person components were regressed on the within-person components of both constructs at the immediately preceding wave, yielding an autoregressive path for each construct and two cross-lagged paths, GAWR(t-1) → LCWSE(t) (the erosion pathway, H2) and LCWSE(t-1) → GAWR(t) (the compensation pathway, H3). The within-person components at Wave 1 were allowed to correlate freely, and within-person residual (co)variances at Waves 2–4 were estimated freely (not constrained equal) in both the constrained and freely estimated models. Autoregressive and cross-lagged paths were constrained equal across the three wave-to-wave transitions (a time-invariance constraint standard in RI-CLPM applications with more than three waves): each of the four path types (2 autoregressive, 2 cross-lagged) was estimated once instead of three times, which is why the constrained model gains exactly 8 degrees of freedom (2 fewer parameters × 4 path types) relative to the freely estimated model. We additionally fit a partially invariant model in which the four structural paths spanning the Wave 2 to Wave 3 transition were freed while the remaining two transitions stayed constrained, which tests whether the longer summer interval carries a different lagged process from the two term-time intervals.

**Estimation.** Models were estimated in R 4.6.1 with the lavaan package (version 0.7–2) [45], using full-information maximum likelihood on all 387 baseline respondents. An initial implementation in Python (semopy 2.3) freely estimated a residual variance for each observed composite score alongside the wave-specific within-person component. Re-estimating that specification in lavaan established that the two sets of terms are not separately identified: its expected information matrix is rank-deficient (smallest eigenvalue 1.1 × 10 ^−^ ^15^, condition number 2.8 × 10^17^), and the Wave-1 pair trades off directly, with the residual variance of the GAWR composite estimated at −6.10 (*SE* = 71.30) against a within-person variance of 6.65 (*SE* = 71.28) for the same wave. This is the source of the instability that appeared under FIML; it is a property of the specification, reproduces across both software packages, and is not a limitation of either. Fixing the composite-score residual terms to zero, as the measurement model above specifies, and constraining the random intercepts orthogonal to the Wave-1 within-person components yields a well-conditioned model (condition number 78) in which all estimates are admissible and FIML converges on the full baseline sample. Every result reported below uses this specification. Model fit was evaluated using the comparative fit index (CFI) and Tucker-Lewis index (TLI), with values above .95 indicating good fit, the root mean square error of approximation (RMSEA), with values below .06 indicating good fit, and the standardized root mean square residual (SRMR), with values below .08 indicating good fit.

**Testing H4.** We compared the magnitude of the two cross-lagged paths in two complementary ways. First, we fit a constrained model in which the erosion and compensation paths were set equal to one another and compared its fit to the freely estimated model using a chi-square difference test; a significant difference indicates the two paths are not equal in the population. Second, because a single equality test can be sensitive to sampling variability with a moderate sample size, we conducted a nonparametric bootstrap (5,000 resamples drawn with replacement from the complete-case sample, so that every resample supports the same model), refitting the freely estimated model in each resample and recording the difference between the absolute magnitudes of the two unstandardized cross-lagged paths, yielding an empirical sampling distribution and a 95% percentile confidence interval for this difference.

**Post hoc power analysis.** Because a null or marginal result is uninformative unless the design had a realistic chance of detecting the effect in question, we conducted a Monte Carlo power analysis after fitting the primary model, using the estimated parameters (Table 1) as population values. We simulated 2,000 datasets at the analytic sample size (*N* = 387) and a further 2,000 at the complete-case size (*N* = 298), refit the RI-CLPM structural model to each, and computed the proportion of replications in which each parameter of interest (the H1 random-intercept covariance, the H2 erosion path, the H3 compensation path, and the H4 equality comparison) reached *p* < .05. To supply the sample-size context the design lacked in advance, we repeated the simulation from *N* = 150 to *N* = 1,200 with 1,000 replications at each sample size, yielding a sensitivity curve for each parameter. These estimates are used to interpret the null and marginal results in the Results and Discussion.

## 4. Results

### 4.1 Descriptive statistics and preliminary checks

Table 3 presents means, standard deviations, and zero-order correlations for GAWR and LCWSE at each wave. GAWR composite means ranged from 2.96 to 2.97 across waves (*SD* ≈ 0.81–0.84) and LCWSE composite means ranged from 3.21 to 3.23 (*SD* ≈ 0.89–0.90), indicating no evident floor or ceiling effects. Concurrent (same-wave) correlations between GAWR and LCWSE were negative at every wave, ranging from −.24 to −.33, consistent with the general theoretical expectation that reliance and confidence move in opposite directions, though zero-order correlations of this kind cannot distinguish between-person from within-person sources of covariation, which is precisely the question the RI-CLPM was designed to address.

**Table 3:** Means, standard deviations, and zero-order correlations among study variables (N  =  387; pairwise-present)).

Variable	M	SD	1	2	3	4	5	6	7	8
1. GAWR W1	2.96	0.84	—							
2. GAWR W2	2.96	0.83	.57	—						
3. GAWR W3	2.97	0.81	.44	.59	—					
4. GAWR W4	2.96	0.82	.36	.45	.60	—				
5. LCWSE W1	3.22	0.90	−.24	−.35	−.27	−.21	—			
6. LCWSE W2	3.21	0.90	−.23	−.29	−.38	−.32	.54	—		
7. LCWSE W3	3.23	0.89	−.18	−.23	−.33	−.35	.39	.63	—	
8. LCWSE W4	3.22	0.89	−.17	−.18	−.27	−.32	.35	.48	.63	—

*Note. GAWR = Generative AI Writing Reliance; LCWSE = Literary Creative Writing Self-Efficacy; W = Wave. Statistics are computed on all available observations at each wave (n = 387, 356, 330, and 298 at Waves 1–4 respectively); correlations use pairwise-present cases. Critical r at p < .01 (two-tailed) is approximately .149 at n = 298 and .132 at n = 387; all reported correlations of |r| ≥ .17 exceed both values. Cronbach's α ranged from .930 to .935 for GAWR and from .946 to .952 for LCWSE across the four waves; McDonald's ω ranged from .933 to .937 and from .948 to .954 respectively.*

The Wave 1 measurement model, specifying separate GAWR and LCWSE factors, fit the data well, χ²(64) = 78.58, *p* = .104, CFI = .997, TLI = .996, RMSEA = .024, supporting the use of composite scores in the structural analysis that follows. Standardized item loadings at Wave 1 ranged from .80 to .90 for GAWR and from .82 to .89 for LCWSE, with no item loading below .80 (Table 4). A parcel-based test of measurement invariance across all four waves in the complete-case sample (*n* = 298) found that constraining loadings to equality (metric invariance) did not significantly worsen fit relative to the freely estimated (configural) model, Δχ²(6) = 4.38, *p* = .625, ΔCFI = +.0004, supporting the assumption that GAWR and LCWSE were measured comparably at every wave.

**Table 4:** Wave-1 standardized item loadings (N  =  387)).

GAWR item	Loading	LCWSE item	Loading
1	.81	1	.85
2	.80	2	.83
3	.81	3	.87
4	.90	4	.88
5	.87	5	.89
6	.85	6	.87
—	—	7	.82

*Note. All loadings statistically significant, p < .001. Model: two-factor CFA, Wave 1, N = 387.*

Two checks addressed common method bias beyond the procedural safeguards described in Methods. First, a single-factor model in which all Wave-1 GAWR and LCWSE items loaded on one common factor fit dramatically worse than the two-factor model, χ²(65) = 2398.15, CFI = .460, RMSEA = .305, providing evidence against a pervasive common-method factor. Second, we compared Wave-1 GAWR and LCWSE scores between the 298 participants retained through Wave 4 and the 89 who dropped out before completing all four waves; neither GAWR (*M*_completers_ = 2.97 vs. *M*_dropouts_ = 2.94, *t* = 0.29, *p* = .771) nor LCWSE (*M*_completers_ = 3.23 vs. *M*_dropouts_ = 3.20, *t* = 0.33, *p* = .743) differed between completers and dropouts, indicating attrition was not related to baseline levels of either construct. Attrition was examined on two further fronts. Completers and non-completers did not differ on any of the five Wave-1 covariates (gender, *t*(144.4) = 0.23, *p* = .822; academic year, *t*(137.6) = −1.04, *p* = .302; literature GPA, *t*(149.1) = 1.09, *p* = .279; AI familiarity, *t*(156.6) = 0.35, *p* = .725; creative-writing course load, *t*(156.9) = 0.33, *p* = .741), and a logistic regression predicting dropout from all seven Wave-1 variables jointly did not improve on an intercept-only model, χ²(7) = 2.76, *p* = .906, with no individual predictor approaching significance. Because equal baseline levels still leave open the possibility that dropout tracks each construct's rate of change, we fit a further logistic regression among the 356 participants who supplied at least Waves 1 and 2, predicting later dropout from each participant's Wave 1 to Wave 2 change on both constructs. Neither change slope predicted dropout (ΔGAWR, *b* = −0.16, *SE* = 0.22, *p* = .473; ΔLCWSE, *b* = 0.09, *SE* = 0.19, *p* = .633), and adding the two slopes to a model containing baseline levels alone did not improve fit, χ²(2) = 0.81, *p* = .667. Attrition is therefore unrelated to the study's constructs at baseline, to their early trajectories, and to the measured demographics. The primary analysis retains all 387 baseline respondents under FIML in any case, so the question bears on the complete-case robustness check and not on the model we interpret. Wave-1 sample characteristics are reported in Table 2. None of the five covariates was associated with either random intercept: the largest correlation with a random-intercept factor score was .089 (AI familiarity with RI-GAWR, *p* = .079), and regressing each random intercept on all five covariates jointly explained almost no variance (RI-GAWR, *R*² = .014, *F*(5, 381) = 1.05, *p* = .386; RI-LCWSE, *R*² = .009, *F*(5, 381) = 0.67, *p* = .648). The stable between-person components of reliance and confidence are therefore not reducible to the demographic and exposure variables measured here.

### 4.2 RI-CLPM structural model

The time-invariant RI-CLPM (autoregressive and cross-lagged paths constrained equal across the three wave-to-wave transitions) fit the data well under FIML on all 387 baseline respondents, χ²(17) = 7.44, *p* = .977, CFI = 1.000, TLI = 1.016, RMSEA = .000, SRMR = .021. Freeing all twelve structural paths did not improve fit, Δχ²(8) = 4.46, *p* = .814, so the time-invariance constraint costs nothing in fit. Freeing only the four paths that span the Wave 2 to Wave 3 transition, the interval that runs across the summer vacation, likewise did not improve fit, Δχ²(4) = 2.25, *p* = .689; the unstandardized compensation path estimated for the summer transition alone (−.250, *SE* = .061, *p* < .001) sits close to the estimate shared by the two term-time transitions (−.231, *SE* = .046, *p* < .001). The unequal spacing described in Section 3.1 therefore leaves no detectable trace on the lagged process, and the equality constraint is retained for the models reported below. The complete-case model (*n* = 298) fit comparably, χ²(17) = 11.80, *p* = .812, CFI = 1.000, TLI = 1.010, RMSEA = .000, SRMR = .029.

**H1 (trait-level association).** The correlation between the random intercepts of GAWR and LCWSE was negative, as hypothesized, *r* = −.342, but a likelihood-ratio test could not distinguish the underlying covariance from zero, Δχ²(1) = 2.26, *p* = .133 (unstandardized covariance −.073, *SE* = .043). The complete-case estimate was similar in magnitude and similarly imprecise, *r* = −.383, Δχ²(1) = 2.43, *p* = .119. H1 was not supported at conventional significance.

**H2 (erosion pathway).** The within-person path from GAWR to subsequent LCWSE was negative and fell short of the conventional threshold, β = −.085, *SE* = .048, *p* = .072 (likelihood-ratio test Δχ²(1) = 3.19, *p* = .074). In the complete-case subsample the same path crossed the threshold, β = −.100, *SE* = .049, *p* = .037, which is a difference in significance and not in estimated magnitude. H2 was not supported at conventional significance in the primary FIML model; Section 4.3 reports the power this design had to detect an effect of that size.

**H3 (compensation pathway).** The within-person path from LCWSE to subsequent GAWR was substantially larger and statistically significant, β = −.276, *SE* = .050, *p* < .001 (likelihood-ratio test Δχ²(1) = 29.69, *p* < .001). Within-person declines in literary creative writing self-efficacy predicted a subsequent within-person increase in generative AI writing reliance, net of each construct's own prior level and of stable trait differences. The complete-case estimate was effectively identical, β = −.275, *SE* = .051, *p* < .001. H3 was supported.

**H4 (relative pathway magnitude).** The point estimate of the compensation path (|β| = .276) was roughly three times that of the erosion path (|β| = .085). A likelihood-ratio test comparing a model in which the two cross-lagged paths were constrained equal (in signed form, since both paths are negative) to the freely estimated model reached significance, Δχ²(1) = 8.83, *p* = .003. A nonparametric bootstrap (5,000 resamples) yielded a median difference in absolute unstandardized magnitude (|compensation| - |erosion|) of .124, with a 95% percentile confidence interval of [.020, .218] that excluded zero; the compensation path exceeded the erosion path in magnitude in 99.1% of resamples. The complete-case comparison agreed, Δχ²(1) = 6.46, *p* = .011. Because both cross-lagged paths carry the same sign here, the signed-equality likelihood-ratio test and the magnitude-based bootstrap target effectively the same comparison in this dataset, though that correspondence would not hold in general if the two paths had opposite signs. By the .05 threshold specified in advance, H4 was supported; Table 5 reports both tests. What that support licenses is bounded by the power analysis below. The comparison sets a path this design detected in essentially every simulated replication against a path it detected in about half of them, so it establishes an asymmetry in what the design could see and does not establish that compensation is the stronger mechanism as a general matter.

**Table 5:** H4 pathway comparison: Likelihood-ratio test and nonparametric bootstrapbootstrap.

Test	Value
Point estimate, H3 compensation (β)	.276
Point estimate, H2 erosion (β)	.085
Difference (H3 − H2, absolute magnitude)	.191
Likelihood-ratio equality-constrained model comparison, Δχ²(1)	8.83, p = .003
Bootstrap (5,000 resamples), median |H3| − |H2|	.124
Bootstrap 95% percentile CI	[.020, .218]
Proportion of resamples with |H3| > |H2|	99.1%
Complete-case likelihood-ratio comparison (n = 298), Δχ²(1)	6.46, p = .011

*Note. Estimates are from the time-invariant RI-CLPM estimated by FIML on all 387 baseline respondents (* Table 1 *). The point estimates in the first three rows are standardized; the likelihood-ratio comparisons and the bootstrap operate on the unstandardized cross-lagged coefficients (FIML: compensation −.239, erosion −.098; complete case: −.238 and −.113, an absolute difference of .125). Both the likelihood-ratio test and the bootstrap interval cross the p < .05 / CI-excludes-zero threshold specified in advance, and H4 was supported. Bootstrap resamples were drawn with replacement from the complete-case sample so that each resample supports the same model; all 5,000 converged. See Results 4.2–4.3 for the power-based bounds on what this comparison licenses.*

Autoregressive paths were positive and significant for both constructs (GAWR: β = .324, *SE* = .056, *p* < .001; LCWSE: β = .421, *SE* = .054, *p* < .001), indicating moderate within-person carryover from each wave to the next over and above stable trait differences. Both random-intercept variances were significant (Var(RI-GAWR) = .216, *SE* = .048, *p* < .001; Var(RI-LCWSE) = .211, *SE* = .063, *p* < .001), and the within-person residual variances at Waves 2–4 were each estimated with a standard error roughly an order of magnitude smaller than the estimate itself. The imprecision that Lüdtke and Robitzsch [33] and Zheng and Valente [34] associate with RI-CLPM at a limited wave count is concentrated in one quantity here, the random-intercept covariance tested as H1, which remains the least precisely estimated parameter in the model and the one the power analysis below identifies as the hardest to detect. Table 1 reports the complete set of standardized structural parameters.

### 4.3 Post Hoc Power Analysis

Because H1 and H2 were both null at conventional thresholds, and because H4 compares one clearly significant path with one non-significant path, we conducted a post hoc Monte Carlo power analysis to establish what this design could detect. Using the estimated parameters from the model above as population values, we simulated 2,000 datasets at *N* = 387 and refit the RI-CLPM to each. Empirical power (the proportion of replications reaching *p* < .05) was 100.0% for H3's effect size (β = −.276), 91.4% for the H4 equality comparison, 53.2% for H2's effect size (β = −.085), and 44.2% for H1's effect size (*r* = −.342). At the complete-case size of *N* = 298 the corresponding values were 99.9%, 83.0%, 44.4%, and 39.3%. With 2,000 replications the binomial standard error on each estimate is under 1.2 percentage points, so the ordering among them is not an artifact of simulation noise. This design was well powered to detect the compensation path and the pathway comparison, and had close to even odds of detecting the erosion path and the trait-level covariance at the magnitudes estimated here. We read the H1 and H2 null results accordingly: as weak evidence about whether those associations exist, and not as evidence against them.

The sensitivity analysis across sample sizes gives the same conclusion a scale. Repeating the simulation from *N* = 150 to *N* = 1,200 with 1,000 replications at each sample size yields the power curve reported in Table 6. The compensation path (H3) is detected in more than nine replications in ten from *N* = 150 upward, so no plausible sample size available to this design would have left H3 undetected. The erosion path at the magnitude estimated here needs a sample of roughly 700 to reach 80% power, and the trait-level covariance needs a sample of roughly 1,000; both exceed what a four-wave panel of this kind can realistically retain. This is the sample-size context the study lacked in advance, and it identifies the erosion pathway and the trait-level association as the two quantities that a replication would need to be designed around.

**Table 6:** Monte carlo power to detect each parameter, by sample size.

N	H1 trait covariance (r = −.342)	H2 erosion (β = −.085)	H3 compensation (β = −.276)	H4 pathway comparison
150	28.8	27.7	96.1	53.7
200	29.6	34.0	99.6	63.3
250	34.9	38.2	99.6	76.8
298 (complete-case sample)	39.3	44.4	99.9	83.0
350	47.8	49.4	100.0	87.0
387 (analytic sample)	44.2	53.2	100.0	91.4
400	47.7	56.4	100.0	91.6
500	54.2	62.2	100.0	96.3
600	60.7	70.1	—	—
700	62.7	80.9	—	—
800	75.1	83.8	—	—
900	78.9	88.2	—	—
1,000	80.5	90.2	—	—
1,200	87.3	95.0	—	—

*Note. Entries are empirical power, the percentage of simulated replications in which the parameter reached p < .05. Population values were the parameter estimates of the time-invariant RI-CLPM reported in* Table 1*. Replications: 2,000 at N = 298 and N = 387, 1,000 at every other sample size, and 1,000 at N = 600 and above for H1 and H2 only (entries left blank for H3 and H4, which were already at ceiling). Binomial standard error is at most 1.6 percentage points for the 1,000-replication rows and 1.2 points for the 2,000-replication rows.*

### 4.4 Summary of hypothesis tests

H3 and H4 were supported. H1 and H2 were not supported at the *p* < .05 threshold specified in advance. The power analysis indicates that this design was well powered for H3 and for the H4 comparison and only moderately powered for H1 and H2, so the contrast between the two cross-lagged paths reflects both a difference in estimated magnitude and a difference in what this design could detect. We treat H3 as the paper's central empirical finding, H4 as a statement about relative magnitude within the range this design could resolve, and H1 and H2 as questions these data leave open.

## 5. Discussion

### 5.1 Overview of findings

This study asked a question that cross-sectional and standard longitudinal designs cannot answer on their own: when Chinese Language and Literature undergraduates’ generative AI writing reliance and literary creative writing self-efficacy move together over an academic year, which one is more reliably driving the other? Using a four-wave random-intercept cross-lagged panel model estimated by full-information maximum likelihood on all 387 baseline respondents, we found a within-person pathway from declining self-efficacy to subsequent increases in AI reliance that was statistically significant and estimated with essentially complete power (β = −.276, *p* < .001). The reverse pathway, from increased reliance to subsequent declines in self-efficacy, was about a third the size and fell just short of the conventional threshold (β = −.085, *p* = .072). A direct comparison of the two pathways favored compensation by both a likelihood-ratio test (Δχ²(1) = 8.83, *p* = .003) and a 5,000-resample bootstrap whose 95% confidence interval for the difference excluded zero. The trait-level correlation between the two constructs’ stable components was negative and moderate in size (*r* = −.342) but could not be distinguished from zero. Read plainly: H3 and H4 were supported, H1 and H2 were not, and the erosion pathway sits in a region where this design had roughly even odds of detection, which makes its non-significance uninformative about whether the mechanism operates.

### 5.2 What the null results for H1 and H2 can and cannot support

Before drawing any theoretical lesson from the H1 and H2 nulls, it is worth asking what this design could have detected. Taking the estimated parameters as population values and simulating 2,000 datasets at *N* = 387, empirical power to detect the erosion path (H2, β = −.085) at *p* < .05 was 53.2%, and power to detect the random-intercept covariance (H1, *r* = −.342) was 44.2%; the compensation path was detected in 100.0% of replications. A non-significant result from a test with roughly even odds of detection is close to uninformative in either direction. It is not evidence that erosion fails to operate, and we do not present it as such. What it licenses is the narrower statement that an erosion effect of this magnitude, if real, would be detected by this design about half the time, and was not detected here. A cognitive-offloading account in which reliance gradually displaces mastery experience [1–3] remains an open possibility on these data. We are explicit about the asymmetry this creates in the abstract and conclusion as well: the erosion pathway is undetermined here, and no comparison in this paper should be read as showing that erosion is absent.

This also bounds what H4 can support. The compensation path was both larger and the more precisely estimated of the two, and the equality test between them was itself well powered (91.4% at *N* = 387), so the conclusion that the two paths differ in magnitude is on firm footing within this design. The conclusion that compensation is the stronger mechanism in general is not, because the comparison sets an estimate this design could always detect against one it could detect about half the time, and because nothing in the offloading account specifies how much cumulative exposure an erosion effect requires before it becomes visible. A one-year window may simply be shorter than the mechanism needs. The defensible reading is that compensation operates on a timescale this design resolves and erosion, if it operates, does not announce itself within four waves and one academic year.

### 5.3 What would have to be true for the trait-level finding (H1) to be real

The point estimate for the random-intercept correlation (*r* = −.342) was moderate and in the expected direction, and the likelihood-ratio test around it (Δχ²(1) = 2.26, *p* = .133) could not separate it from zero. Lüdtke and Robitzsch [33] and Zheng and Valente [34] have argued that RI-CLPM's random-intercept variance and covariance components are estimated with less precision than its within-person cross-lagged paths when the number of waves is limited, because separating a stable trait from a correlated sequence of state-level deviations requires more repeated observations than comparing two consecutive within-person changes. Our own simulation matches that argument closely: at *N* = 387 the trait-level covariance was the single hardest parameter in the model to detect (44.2% power, against 100.0% for the compensation path), and the sensitivity curve in Table 6 puts the sample size needed for 80% power on this parameter well beyond what a four-wave design of this kind retains. The substantive question H1 poses, whether chronically more AI-reliant students are also chronically less confident, is answerable in principle; it is not answerable at this wave count and this N, and we report the estimate as directional evidence awaiting a design built for it.

### 5.4 Practical implications

The practical question institutions are actually facing is concrete: should a Chinese Language and Literature program spend its limited intervention budget on restricting or monitoring AI access in creative writing coursework, or on something else? Our results speak most directly to that choice through H3, the one path estimated with reasonable power and found significant. A restriction-focused policy implicitly assumes reliance is the causal agent eroding confidence; that is the pathway (H2) this design could detect only about half the time and did not find at conventional significance. The pathway we did find, with adequate power, ran the other way: from lower confidence to higher subsequent reliance.

Two concrete implications follow. First, the compensation path was detectable across the intervals this design used, approximately nine weeks within a semester and approximately 21 weeks across the summer, and freeing the longer interval did not change the estimate. A meaningful amount of time can therefore elapse between a dip in confidence and the increase in reliance it predicts, and the relationship survives a break in instruction. This argues for front-loading confidence-focused intervention early in a course rather than waiting for a midterm check-in; we do not have the within-wave resolution to say how many weeks the lag actually takes for a given student, only that it operates on a timescale the model detects at intervals of nine weeks and longer, which is consistent with earlier being better than later. Second, “building confidence” is vague enough to be useless as a recommendation unless tied to Bandura's [2] and Pajares's [3] own account of where self-efficacy comes from: mastery experience specifically, not general encouragement. In practice, this means low-stakes, ungraded opportunities to complete a full unassisted draft early in the term, with feedback that names what the student did well in the parts they wrote themselves, in place of generic praise or generic reassurance that “AI is just a tool.” The instructional-modality literature gives this recommendation a more concrete anchor than our own data can. Talebzadeh et al. [21] found writing self-efficacy trajectories to differ across flipped, online, and in-person delivery of the same writing instruction, which implies that the delivery format of an early-semester confidence intervention is itself a design variable and not a neutral container. Ahangaran and Pouyan [22] show that automated corrective feedback can be integrated into writing instruction without displacing the learner's own production, which is the distinction our reliance construct turns on: feedback on work the student has already written leaves the mastery experience intact, whereas delegation of the generative act does not. A program acting on our findings would therefore separate the two uses in its own policy language, and route the AI budget toward feedback on student-produced drafts.

We are not recommending that institutions ignore how much students rely on AI in literary coursework. We are saying that, on the evidence here, the more promising point of intervention is the confidence deficit that precedes heavier reliance, not the reliance itself.

### 5.5. Limitations

Several limitations qualify these conclusions. First, this design's power to detect the erosion pathway at the magnitude estimated here was 53.2%, so the four-wave, one-year window cannot adjudicate between “erosion does not operate” and “erosion operates too slowly, or too weakly, for this design to see it.” Distinguishing those possibilities requires either a longer panel or a substantially larger sample, not a different interpretation of the same data. Second, both constructs were measured exclusively via self-report; we followed procedural safeguards against common method bias [43,44] and additionally ran a single-factor comparison model as a baseline check, but self-reported reliance and self-reported confidence still share a measurement source that no procedural safeguard fully removes, and future work would benefit from behavioral or trace-data measures of actual AI use alongside self-report. Third, the sample was drawn from Chinese Language and Literature programs specifically; generalization to other creative writing contexts should be made cautiously given how culture- and genre-specific our operationalization of literary creative writing self-efficacy was. Fourth, we did not measure the type or sophistication of AI tool used, and a tool used for grammar-level assistance plausibly carries different implications than one used to generate entire passages of original prose; future work distinguishing levels of AI delegation within the broader reliance construct would sharpen these claims. Fifth, the composite measures for GAWR and LCWSE were assembled from items adapted across several source instruments in place of a single validated scale adopted wholesale. Section 3.3 sets out the criterion each item had to meet, the Wave-1 standardized loadings (Table 4) were uniformly high, and both α and ω exceeded .93 at every wave, but internal consistency and single-occasion factor structure are not construct validation. Neither instrument has been validated against external criteria or in an independent sample, and both should be treated as study-specific composites until that work is done. Sixth, two of the cited sources [30,33] are unreviewed preprints rather than peer-reviewed publications, reflecting how recent this literature is; this is a limitation of the evidence base this study draws on, not only of the study itself, and readers should weight those specific claims accordingly until published versions are available.

Seventh, the analysis was not preregistered. The four directional hypotheses were derived from the two theoretical accounts before the data were analyzed, and each specifies an expected sign, but no timestamped analysis plan exists that would let a reader verify the ordering independently. The distinction between the confirmatory and exploratory portions of this paper therefore rests on our account of it, and H4 in particular was framed as a hypothesis while carrying an acknowledged asymmetry in power between the two paths it compares.

### 5.6. Future directions

The most direct extension of this work is a longer panel, adequately powered to detect a small erosion effect if one exists, ideally spanning multiple academic years. The sensitivity curve in Table 6 puts that requirement at roughly 700 participants for the erosion path and roughly 1,000 for the trait-level association at the magnitudes estimated here, which is a specification a multi-site collaboration could meet. A second direction would examine moderators of the compensation pathway: if declining confidence drives reliance, which students are most susceptible matters for targeting intervention, for instance students with higher trait anxiety, lower baseline literary self-concept, or less prior exposure to low-stakes writing practice. Third, replication with a larger sample and, where feasible, more than four waves would give the trait-level question (H1) a fairer test than the one we were able to run.

## 6. Conclusion

This study asked whether generative AI writing reliance erodes literary creative writing self-efficacy, or whether declining self-efficacy drives increased reliance, among Chinese Language and Literature undergraduates tracked across one academic year. A four-wave random-intercept cross-lagged panel design, estimated by full-information maximum likelihood on all 387 baseline respondents, separated these two directional accounts from the stable trait-level association between them. The compensation pathway, from declining confidence to subsequent increased reliance, was significant and detected in essentially every replication of a Monte Carlo power simulation. The erosion pathway, from increased reliance to subsequent declining confidence, did not reach conventional significance, and the same simulation put this design's power to detect an effect of the magnitude estimated for it at 53.2%. The formal comparison between the two pathways favored compensation on both a likelihood-ratio test and a bootstrap interval that excluded zero.

We want to be exact about what that comparison does and does not show. It establishes that the two pathways differ in magnitude within the range this design could resolve. It does not establish that compensation dominates erosion, and the non-significant erosion path is not evidence that erosion is absent: at roughly even odds of detection, that null carries no weight against the mechanism, and whether reliance erodes confidence over a longer horizon remains an open question that these data were not able to close. For Chinese Language and Literature programs deciding how to respond to generative AI in creative writing coursework, the finding that does carry weight points toward a specific choice: building students’ confidence and mastery experience in unassisted composition through low-stakes early-semester practice addresses the mechanism this study established, more directly than restricting AI access on its own would.

More broadly, the results here illustrate a point that matters for how the field studies generative AI's effects on skill and confidence. A null cross-lagged path in a short panel is not evidence that a mechanism does not operate, and treating it as such risks foreclosing questions that a better-powered design would still need to ask. The same caution applies to the estimation layer beneath the null. The instability we initially encountered when retaining all baseline cases proved to be a property of a specification and not of a software package, and it was diagnosable only by refitting the model in a second, independently implemented estimator. Reporting a power analysis alongside a null result, and checking a fragile-looking solution against a second platform before interpreting it, are two small methodological habits that would let this literature accumulate honestly instead of mistaking “not yet detected” for “not real.”

## Supporting information

S1 File Survey instruments. Full item wordings for the Generative AI Writing Reliance (GAWR) scale and the Literary Creative Writing Self-Efficacy (LCWSE) scale, including the adaptation notes described in Section 3.3.
(DOCX)

S2 File Anonymized dataset. Item-level responses of all 387 baseline participants to the six GAWR and seven LCWSE items at each of the four waves, with the five Wave-1 covariates. A codebook sheet defines every variable and its coding; empty cells mark waves a participant did not complete.
(XLSX)

S3 File Analysis code. R scripts (R 4.6.1, lavaan 0.7–2) that reproduce the reported statistics from S2 File, a README that maps each table and in-text result to the script producing it, and the output of each script.
(ZIP)
