# Supplementary material for: Does low confidence drive reliance on AI, or does reliance erode confidence? A four-wave RI-CLPM study of chinese language and literature undergraduates
Source: PLoS One. 2026 Oct 26;21(10):e0361489. doi: 10.1371/journal.pone.0361489 (PMC13704868; doi:10.1371/journal.pone.0361489)
Supplement: Supplementary file 1 [file pone.0361489.s001.docx]

**S1 File. Survey Instruments**

This file presents the full item wordings for the two focal measures used in this study. Both scales used a 5-point Likert response format (1 = strongly disagree, 2 = disagree, 3 = neither agree nor disagree, 4 = agree, 5 = strongly agree) and were administered identically at all four waves. Items were presented in Chinese; the English wordings below are back-translations.

## Generative AI Writing Reliance (GAWR)

*Six items adapted from Wei et al. (2026), Akbari et al. (2026), and Özsoy et al. (2026). Items were selected for their emphasis on task delegation and reduced autonomy in production tasks, and reworded to reference literary and creative writing specifically. Items indexing affective attachment, withdrawal symptoms, or loss of control were not carried over (see Section 3.3 for the construct distinction from general AI use and clinical AI dependence).*

**1.** I rely on AI tools to generate ideas for my literary writing assignments.

**2.** When I have a creative writing task, I turn to AI to produce an initial draft or outline before attempting one myself.

**3.** I depend on AI-generated text to supply phrasing or descriptive passages that I incorporate into my literary work.

**4.** Without AI assistance, I would find it difficult to complete my literary writing assignments to the standard I expect.

**5.** I use AI tools to handle parts of the creative writing process that I could do on my own but prefer not to.

**6.** The proportion of my literary writing output that originates from AI rather than from my own composing has increased over time.

## Literary Creative Writing Self-Efficacy (LCWSE)

*Seven items adapted from Pajares (2007), Pajares and Valiante (1997), Pajares et al. (2006), Graham and MacArthur (1988), Locke and Johnston (2016), and Doenyas et al. (2023). Each item was required to ask about confidence in executing a self-generated writing task; items concerning writing outcomes, self-regulation, or the teaching of writing were excluded. All items were rewritten to name a literary genre (poetry, prose, or short fiction) in place of the academic genre of the source instrument (see Section 3.3).*

**1.** I am confident I can write an original short story that expresses my own voice.

**2.** I am confident I can compose a poem that conveys a mood or image I have in mind.

**3.** I am confident I can develop a compelling narrative arc for a piece of fiction without outside assistance.

**4.** I am confident I can revise a draft of my own creative prose until it meets my own standards of quality.

**5.** I am confident I can produce a piece of literary writing that is recognizably my own work and not interchangeable with what others would write on the same topic.

**6.** I am confident I can write an original descriptive passage that captures a scene or character as I envision it.

**7.** I am confident I can complete a creative writing assignment from start to finish relying on my own ideas and language.

## Covariates (Wave 1 only)

**Gender.** Self-reported.

**Academic year.** Second, third, or fourth year of undergraduate study.

**Literature GPA.** Self-reported cumulative GPA in literature coursework (4-point scale).

**General AI familiarity.** Single item: "How familiar are you with generative AI tools in general (not limited to writing)?" rated 1 (not at all familiar) to 5 (very familiar).

**Creative-writing course load.** Number of creative writing or literary composition courses taken or currently enrolled in at the time of Wave 1.
